# A Detailed Account of Severe Bell’s Palsy: An Autobiographical Case Report

**DOI:** 10.7759/cureus.19837

**Published:** 2021-11-23

**Authors:** Kyril L Cole, Chad Cole

**Affiliations:** 1 Neurosurgery, University of Utah School of Medicine, Salt Lake City, USA; 2 Neurosurgery, University of New Mexico, Albuquerque, USA

**Keywords:** bell's palsy, bell's palsy mental health, bell, disease management, bell's palsy treatment, bell's palsy diagnosis, severe, facial nerve paralysis, autobiographical case report

## Abstract

Bell’s palsy is a relatively rare neurologic disorder with a limited selection of helpful therapies. This case report describes the author’s initial three-month experience living with severe Bell’s palsy, including a detailed history and timeline of the initial development of symptoms, treatments pursued, and psychological stress during the disease progression. A particular focus is placed on the emotional burden Bell’s palsy can have, exploring possible avenues to improve physician to patient education on mental health and well-being during initial and delayed recovery.

## Introduction

Initially described by Sir Charles Bell in 1821, unilateral Bell’s palsy, also known as “idiopathic facial palsy” or “acute facial palsy of unknown cause,” is a non-progressive neurological disorder of cranial nerve VII, commonly referred to as the “facial nerve” [1]. Symptoms result from swelling and inflammation of the cranial nerve VII. Considered a relatively rare disease, Bell’s palsy affects roughly 40,000 people in the United States every year, showing no sex or racial preference. Similarly, no preference for the left or right side of the face appears to exist [2]. The risk for developing Bell’s palsy can occur at any age, though it is primarily seen in mid to late-life, with the median age of onset at 40 years [3,4].

Bell’s palsy, often characterized by a sudden paralysis of the facial muscles innervated by the facial nerve, commonly results in the temporary weakness of the affected side. While symptoms generally present with unilateral facial weakness, rare occurrences of bilateral facial weakness have been reported. Surprisingly, the true cause of Bell’s palsy remains unclear. Several proposed causes have been examined, with the reactivation of an existing or dormant viral infection as the cause with the most popularity within the scientific community [5,6]. Fortunately, most of those affected fully recover within three-to-six months after initial symptomatic presentation [7].

This case report describes the author’s initial experience living with Bell’s palsy, including detailed descriptions of symptom onset, adaptations, and the often-omitted impact on lifestyle and quality of life. While this account covers only three months, I hope this autobiographical case report provides those afflicted with this neurologic disorder with greater clarity as to what can possibly be expected, while also serving as an additional resource for colleagues managing those with Bell’s palsy. Throughout this case report, “I” will refer to myself, Kyril Cole.

## Case presentation

I was diagnosed with Bell’s palsy in September 2021, at the age of 28 years, with no significant past medical history. I presented to a neurosurgeon with a three-day history of right-sided post-auricular pain, a right-sided cervicogenic patterned headache, and right-sided neck pain initially thought to be occipital neuralgia, in addition to a six-hour history of right-sided facial weakness. On physical examination, I was found to have severe weakness in all facial expressions on the right side of my face, including movements in my forehead, nasal, periorbital, and perioral areas. Based on the acute onset of symptoms and physical findings, I was diagnosed with idiopathic facial palsy and started on prednisone the same day. Before initial symptoms, I took no medications and was current on all vaccinations, including my coronavirus disease 2019 (COVID-19) vaccination. Childhood illnesses were significant for only a varicella-zoster viral infection at the age of five years. I had no past surgical history, head trauma, medication allergies, or family history of Bell’s palsy or other neurological conditions. In terms of social history, at the time of diagnosis, I was a full-time MD/MBA student, married, enjoyed hikes and outdoor activities, and ate an entirely plant-based diet with appropriate vitamin supplementation. Over the past three months since my initial diagnosis, I have pursued all recommended treatments and suggested therapies; however, I have yet to regain any motor function to my right-sided facial muscles. 

Progression of symptoms

My symptoms began three days prior to the facial weakness, which began with a dull ache in and around my right ear, with sharp pain behind my ear, which would radiate down my neck. A headache accompanied this pain with a cervicogenic pattern on the right side of my head. After two days of this pain, I spoke with a colleague who gave a few possible explanations, with occipital neuralgia as the most likely diagnosis given my recent gym workouts. However, I noticed my soup at dinner had a dish-soap taste on the third day of discomfort, which did not agree with occipital neuralgia.

**Figure 1:**
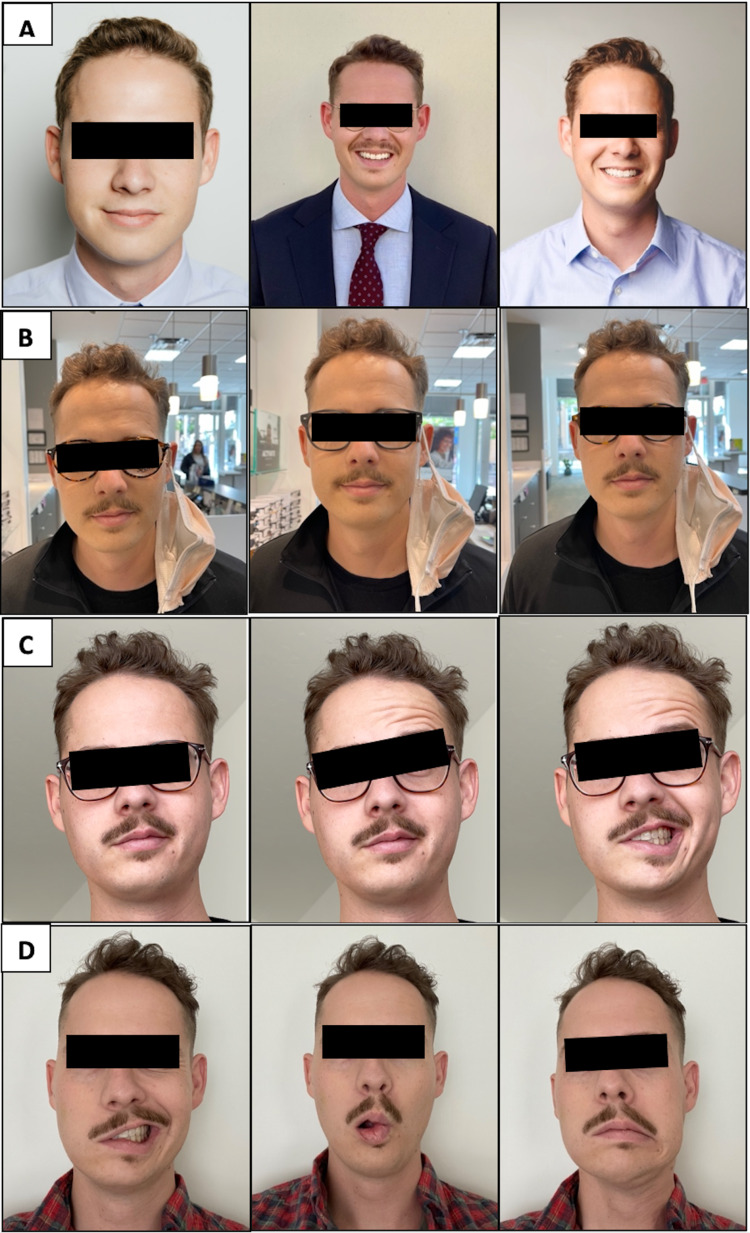
Chronological order of physical changes to facial expressions in progressive Bell's palsy (A) Normal facial expressions before Bell’s palsy demonstrating baseline facial expressions. (B) The first day of symptoms demonstrating diminished ability to smile (first 24 hours). (C) The second day of symptoms demonstrating complete loss of unilateral facial expression (first 48 hours). (D) One month since initial symptoms demonstrating continued complete unilateral facial paralysis.

The following day, I awoke to brush my teeth and use mouthwash, only to find myself spilling mouthwash all over the counter. Upon examination in the mirror, I realized I could no longer fully smile or purse my lips. Additionally, my ability to close my right eye had been severely reduced. During the same day, I confirmed suspicions of Bell’s palsy with a neurosurgeon. I received a prescription for a steroid, beginning within 72 hours of the onset of symptoms as clinically suggested [8]. Throughout the first day of these physical findings, my nerve impairment quickly went from a House-Brackmann scale (HBS) grade III to V over 24 hours. After 48 hours of my symptoms, I had lost all movement to the right side of my face, putting me at a grade VI [9]. A progression of my facial paralysis can be found in Figure 1. 

After 48 hours of the onset of symptoms, a complete understanding of my physical challenges became apparent. These included a rapid onset of right-sided facial paralysis (causing an inability to close my right eye fully, smile, frown, lift my eyebrows, or purse my lips), loss of taste to the front right two-thirds of my tongue, right eye dryness, continued right-sided postauricular pain, drooling in my sleep, paralysis of my right platysma and occipitofrontalis muscles, blockage in my left nasal passageway, and hyperacusis in my right ear.

Treatments pursued

While no effective cure exists for new-onset Bell’s palsy, steroids have shown to be effective at increasing the probability of recovery of facial nerve function [5]. Within 24 hours of my initial diagnosis, I was started on a 14-day 80 mg daily tapered prednisone dose to reduce nerve inflammation and ear pain. However, my symptoms did not improve with prednisone use, and I was subsequently prescribed a seven-day 3 mg oral valacyclovir dose two weeks after my initial diagnosis. During the first four weeks of my facial palsy, I also pursued a once per week acupuncture therapy consisting of 15-20 needles for 60 minutes each session. Lastly, throughout my diagnosis with Bell’s palsy, daily 15-minute facial massages were done to preserve muscle strength, in addition to heavy use of artificial tears to preserve the health of my right cornea and vision overall, as recommended [10].

**Figure 2:**
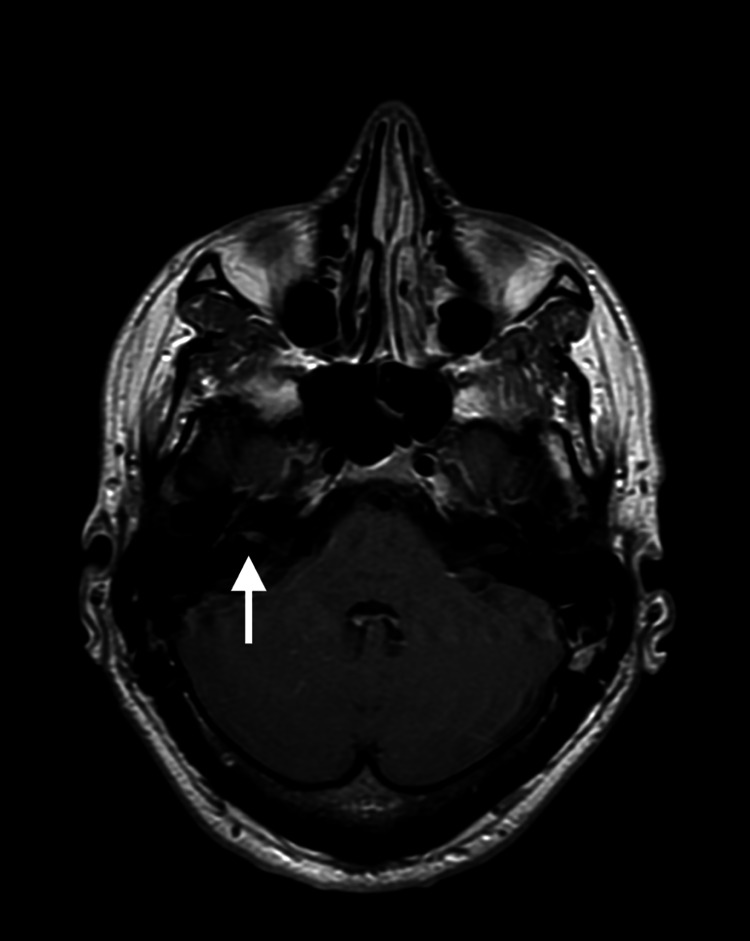
Magnetic resonance imaging (MRI) of the facial nerve Axial T1-weighted postcontrast MRI scan demonstrating enhancement at the right internal acoustic canal (IAC) fundus.

After the prednisone, valacyclovir, and acupuncture therapy had ended, I still had no initial improvement in my symptoms. A magnetic resonance imaging (MRI) scan with and without intravenous contrast was pursued two months after my initial diagnosis to ensure no other pathology was present mimicking the presentation of Bell’s palsy. A T1 image with 15 ml of gadolinium contrast demonstrated enhancement at the right internal auditory canal (IAC) fundus (Figure 2). Fluid-sensitive sequences and other findings revealed normal anatomy, an otherwise unremarkable contrast-enhanced MRI of the brain and cranial nerves at the skull base. Findings were consistent with that of Bell’s palsy or Ramsey Hunt syndrome.

Impact on physical and social activity

Prior to the onset of Bell’s palsy, I was living a hectic yet active lifestyle. This included multiple hikes each week, daily hour-long workout sessions with my wife at the gym, academic and professional courses 60 hours each week, and everyday errands with the family. However, once the symptoms and facial palsy had solidified, I realized just how difficult each of these daily activities would be. Due to my right ear hyperacusis, any loud or high-pitched noise would cause significant pain to my ear. I could no longer go to the gym due to the volume of music, nor go to movies, football games, or bars with family and friends where sounds were far too loud. Even the clanging noises from stacking the cleaned dishes in the cupboard caused me a great deal of ear pain.

Similarly, my right eye no longer produced the tears needed to lubricate my eye, nor could I close my eye sufficiently to keep the moisture in. Studying on the computer screen or watching TV became a significant challenge and strain on my eye, requiring constant artificial tear application. Enjoying a walk outside with even moderate sunlight or wind caused great discomfort. Eating became a significant challenge, as my mouth could not open enough to eat things like sandwiches, pasta, desserts, etc. without making a giant mess and causing me to feel embarrassed. Lastly, sleeping became a difficult challenge, as I would struggle to tape my eyelid closed well enough for a comfortable sleep each night, in addition to my nasal breathing largely blocked off. Through these and other challenges, my illness quickly became an isolating time in my life. 

Impact on psychological wellbeing

Many of those afflicted by Bell’s palsy have significant improvement within three weeks of their initial symptoms, reaching a full recovery within three months [11]. Reading about similar prognoses and expected timelines for recovery gave me a great deal of fear and anxiety for my seemingly nonexistent recovery. I was and continue to be unsure of when or if I will fully recover. These challenges are especially prominent with Bell’s palsy, as it continues to be a poorly understood pathology, with limited resources and medical interventions for assured recovery. This can be a great challenge for patients struggling with Bell’s palsy, as medical professionals are limited in what they can do to improve patient outcomes.

Another feature of Bell’s palsy often overlooked is the impact the physical changes to the face can have on a patient’s psychological well-being. Initially, my new physical appearance was difficult for me, as I could not laugh or express my feelings through animated facial expressions. My appearance was difficult to contemplate, as any emotion was removed from one side of my face, and the other side overcompensated, causing me to look nothing like my original self. Personally, this made online meetings and courses challenging during the COVID-19 pandemic, as it is unprofessional not to show your face on camera. However, it is emotionally taxing to explain to each person what has happened to my face. The challenges of my physical appearance also transitioned to physical encounters, as many would stare and attempt to figure out what is wrong with my face, or they would ask if I had a stroke, dental work, or any facial trauma. These encounters made me want to remove myself and not interact with others, furthering the depression caused by my other social and physical limitations. However, I have become more comfortable with my appearance and understanding of how best to approach others with time.

## Discussion

This case report describes many of the facial palsy-related challenges identified in the literature, including unilateral facial paralysis or increased weakness, hyperacusis, loss of taste, dry eyes with absent tear production, drooling, difficulty eating or drinking, loss of typical facial expressions, headaches, inability to close the eye on the involved side, absent corneal reflex, eye irritation, post-auricular pain, inner ear pain, in addition to unilateral occipitofrontalis and platysma muscle paralysis [10]. While symptoms can vary slightly between cases, most of those with Bell’s palsy, roughly 70% of patients, will spontaneously recover with or without treatment [5]. The Copenhagen Facial Nerve Study evaluated 2570 persons with untreated facial nerve palsy, finding 71% of patients recovering full pre-symptom function and 4% with severe sequelae [12]. To my knowledge, no study has assessed the severity of initial Bell’s palsy presentation and functional recovery, though delayed recovery is often accompanied by some form of abnormal facial function [7]. 

Studied risk factors that put the patient at higher risk for developing Bell’s palsy include a family history of Bell’s palsy, current or recent pregnancy, Lyme disease, head trauma, diabetes, upper respiratory infections, or past viral infections (including herpes simplex virus type 1 and varicella-zoster virus) [12]. Interestingly, my case presents with a very severe version of Bell’s palsy without an initial recovery within the first three months of the disorder. Contrary to common risk factors, I am young (28 years old), active (gym and jogging five times per week), and relatively healthy (a plant-based diet with appropriate vitamin supplementation and a body mass index (BMI) of 22.10) without a recent viral infection. One commonly proposed idea is the reactivation of a dormant virus during an immunosuppressed state due to high stress [5]. Tseng et al. found anxiety disorders to significantly increase the risk of developing Bell’s palsy, including a bidirectional temporal association between the two disorders [13]. During the days and weeks preceding my initial symptoms, I was passing through a stressful time in life, with heavy physical and mental work, high anxiety with professional obligations, and very little sleep in between. My medical history of a varicella-zoster viral infection and the acute stress preceding my symptoms can support such a claim, given that I have no other known risk factors.

Though not curative, several treatment options have been studied to help relieve the symptoms associated with Bell’s palsy. Kim et al. performed a retrospective study on 1710 Bell’s palsy patients, comparing the effectiveness of steroids plus antiviral agents vs. steroids alone in treating severe Bell’s palsy patients over 40 years old. Subgroup analysis showed the combination therapy resulted in significantly higher recovery rates than steroids alone (77.5% vs. 64.1%, p=0.023) [14]. A randomized controlled trial by Shahidullah et al. found similar results, preferring antiviral and steroid combination therapy for improved recovery after Bell’s palsy [15]. While large studies have demonstrated the effectiveness of combination therapies in severe cases of Bell’s palsy, my case was unique because I have had no response to treatment thus far. I took the prescribed prednisone within 72 hours of my onset of symptoms, in addition to valacyclovir two weeks after symptom onset. As my case has been unresponsive to medical intervention and my face continues to be paralyzed, I have an increased risk for incomplete recovery [15].

Medical professionals are proficient at describing the pathophysiology of the neurologic disorder, current best treatments to reduce nerve inflammation, and the limitations in knowledge of the cause. However, in my experience, physicians do not mention the emotional toll that Bell’s palsy can have on one’s life, especially in cases of severe Bell’s palsy (HBS - V, VI) lasting over three months. Sugiura et al. looked at the stress levels of Bell’s palsy patients from initial presentation through full recovery. They found the psychological stress response score of patients with facial palsy to be high on the first hospital visit, suggesting the importance for physicians to treat the psychological aspects of Bell’s palsy [16]. To help patients work through the mental challenges associated with Bell’s palsy, it is critical that physicians provide recommendations for therapists or emotional guidance during this period. Special attention on this subject should be given to patients belonging to the “severe” Bell’s palsy subgroup.

The limitation of this case report is that I can report only the first three months of my experience with Bell’s palsy, given the timeline of the requested autobiographical reports and the recency of my Bell’s palsy onset. A longer timeline with a full recovery and reviewed response to physical therapy would benefit such a case series. A strength of this case report is my detailed description of each portion of my experience thus far, including an in-depth look at the timeline of symptom onset, medications and therapies utilized with their timelines, and the images demonstrating the physical changes in my facial paralysis. Another strength of this case report is the explanation of the emotional toll Bell’s palsy can have on everyday life, something that is often overlooked in medical reports [3-5].

## Conclusions

This autobiographical case report illustrates the typical initial progression of Bell’s palsy symptoms, with the addition of a detailed timeline and description of each step in the progression of my facial palsy. Apart from the various medical articles on Bell’s palsy, few pay particular attention to the emotional, physical, and social toll the disorder can put on the affected individual’s life. Specifically, those who suffer from severe Bell’s palsy, such as my case, would benefit significantly from initial explanations and recommendations on psychological well-being from their treating physician. Consultation with psychologists, psychiatrists, therapists, or other emotional guidance counselors should be encouraged for those affected by delayed recovery from Bell’s palsy. This approach could reduce overall stress from the disorder and potentially assist in hastening any delayed recovery.
